# USING THE ELECTRONIC MEDICAL RECORD OF NURSING HOME RESIDENTS FOR IMPROVED MEASURE OF BEHAVIORAL SYMPTOMS

**DOI:** 10.1093/geroni/igad104.0429

**Published:** 2023-12-21

**Authors:** Hyunkyung Yun, Ellen McCreedy, Vincent Mor, Susan Mitchell

**Affiliations:** Brown University, Providence, Rhode Island, United States; Brown University School of Public Health, Providence, Rhode Island, United States; Brown University School of Public Health, Providence, Rhode Island, United States; Hebrew SeniorLife, Boston, Massachusetts, United States

## Abstract

Behavioral symptoms as study outcomes are measured by routinely collected data such as the Minimum Data Set (MDS), the standardized assessment of nursing home (NH) residents. However, MDS under-detects neuropsychiatric symptoms in people with dementia, and better identification in this outcome measurement is needed. This work aims to determine if the electronic medical record (EMR) NH data, the Interventions to Reduce Acute Care Transfers (eINTERACT) form, could improve the identification of agitated behaviors (behaviors). 77,936 assessments of the MDS and the EMR of 19,705 long-stay residents with dementia from 322 NHs, conducted between January 2020 and August 2022, were used. MDS behaviors were defined as any verbal or physical behaviors directed at others, resistance to care, or other behaviors (e.g., pacing). eINTERACT behaviors were defined as any general agitation or verbal or physical aggression. 96% of NHs used eINTERACT forms for at least half of their residents. On average, 14.8% (SD 35.6) of residents with a qualifying assessment in a given month had an MDS-documented behavior, and 2.4% (SD 15.2) had an eINTERACT-documented behavior. 1.4% (SD 11.7) had behaviors documented only on the eINTERACT form representing about a 10% increase in detectable behaviors in a given month over MDS alone. Results were robust to stratification by NH-level prevalence of MDS behaviors. Using the eINTERACT forms in addition to the MDS behavioral items increased in detection among long-stay residents with ADRD. There is some indication that greater use of eINTERACT is associated with greater increases in behavior identification.

